# MSM Enhances GH Signaling via the Jak2/STAT5b Pathway in Osteoblast-Like Cells and Osteoblast Differentiation through the Activation of STAT5b in MSCs

**DOI:** 10.1371/journal.pone.0047477

**Published:** 2012-10-11

**Authors:** Youn Hee Joung, Eun Joung Lim, Pramod Darvin, So Chung Chung, Ju Woong Jang, Kyung Do Park, Hak Kyo Lee, Heui Soo Kim, Taekyu Park, Young Mok Yang

**Affiliations:** 1 Department of Pathology, School of Medicine, Institute of Biomedical Science and Technology, Konkuk University, Seoul, Republic of Korea; 2 Department of Pediatrics, School of Medicine, Konkuk University, Seoul, Republic of Korea; 3 Korea Bone Bank Co, Ltd. Tower-9, 345-30, Gasan-Dong, Geumcheon-Gu, Seoul, Republic of Korea; 4 Genomic Informatics Center, Hankyong National University, Anseong, Republic of Korea; 5 Department of Biological Sciences, College of Natural Sciences, Pusan National University, Busan, Republic of Korea; 6 Department of Biotechnology, College of Biomedical and Health Science, Konkuk University Glocal Campus, Chung-Ju, Republic of Korea; Inserm U606 and University Paris Diderot, France

## Abstract

Methylsulfonylmethane (MSM) is a naturally occurring sulfur compound with well-known anti-oxidant properties and anti-inflammatory activities. But, its effects on bone are unknown. Growth hormone (GH) is regulator of bone growth and bone metabolism. GH activates several signaling pathways such as the Janus kinase (Jak)/signal transducers and activators of transcription (STAT) pathway, thereby regulating expression of genes including insulin-like growth factor (IGF)-1. GH exerts effects both directly and via IGF-1, which signals by activating the IGF-1 receptor (IGF-1R). In this study, we investigated the effects of MSM on the GH signaling via the Jak/STAT pathway in osteoblasts and the differentiation of primary bone marrow mesenchymal stem cells (MSCs). MSM was not toxic to osteoblastic cells and MSCs. MSM increased the expression of GH-related proteins including IGF-1R, p-IGF-1R, STAT5b, p-STAT5b, and Jak2 in osteoblastic cells and MSCs. MSM increased IGF-1R and GHR mRNA expression in osteoblastic cells. The expression of MSM-induced IGF-1R and GHR was inhibited by AG490, a Jak2 kinase inhibitor. MSM induced binding of STAT5 to the IGF-1R and increased IGF-1 and IGF-1R promoter activities. Analysis of cell extracts by immunoprecipitation and Western blot showed that MSM enhanced GH-induced activation of Jak2/STAT5b. We found that MSM and GH, separately or in combination, activated GH signaling via the Jak2/STAT5b pathway in UMR-106 cells. Using siRNA analysis, we found that STAT5b plays an essential role in GH signaling activation in C3H10T1/2 cells. Osteogenic marker genes (ALP, ON, OCN, BSP, OSX, and Runx2) were activated by MSM, and siRNA-mediated STAT5b knockdown inhibited MSM-induced expression of osteogenic markers. Furthermore, MSM increased ALP activity and the mineralization of MSCs. Taken together, these results indicated that MSM can promote osteogenic differentiation of MSCs through activation of STAT5b.

## Introduction

Growth hormone (GH) and insulin-like growth factor (IGF)-1 are important regulators of bone homeostasis and are central for achieving normal longitudinal bone growth and bone mass [1]. During the prepubertal period, GH and IGF-1 are determinants of longitudinal bone growth, skeletal maturation, and acquisition of bone mass, whereas in adults they are important in the maintenance of bone mass [2], [3]. Bone, a highly mineralized tissue, is delicately regulated by a balance between bone resorption and bone formation. Because osteoblasts originate from mesenchymal stem cells (MSCs), promoting or inhibiting MSCs into an osteoblast lineage is an important step during new bone formation [4], [5]. GH is known to play a role during this process. Due to limitations in GH supply, a limited number of animal and clinical studies were performed until the mid 1980s when recombinant human GH became available. The initial use of recombinant human GH was restricted to treatment of growth-retarded GH-deficient (GHD) children. However, it is now well established that GH also exerts important effects in adults, and GH treatment of adults with GHD is now approved in several countries [6]. Recent studies in both animals and humans have demonstrated that GH exerts potent effects on bone remodeling [6], but recombinant human GH is very expensive. Therefore, there is an increasing need for safer therapeutic agents with efficacy comparable to commercially available drugs for treating bone remodeling disorders.

GH signaling via its receptor is mediated through cascades of protein phosphorylation resulting in activation of nuclear proteins and transcription factors. The growth hormone receptor (GHR) itself is not a tyrosine kinase [7]. Instead, when GH binds to the GHR, it induces receptor homodimerization and activation of the GHR-associated tyrosine kinase Janus kinase 2 (Jak2) [8], [9]. Jak2 is then phosphorylated and, in turn, phosphorylates the GHR and the signal transducers and activators of transcription (STAT) protein. Upon phosphorylation, the STAT proteins either homodimerize or heterodimerize, translocate to the nucleus, bind to their appropriate DNA response element, and stimulate transcription of GH-regulated genes including IGF-1 [10]. GH exerts its effects both directly and via IGF-1, which signals by activating IGF-1R. IGF-1R is a cell surface receptor that contains intrinsic tyrosine kinase activity within its intracellular domain [11]. GH activates STATs 1, 3, 5a, and 5b [12]. A recent study suggested that the UMR-106 osteoblast-like osteosarcoma cell line expresses a GH-responsive Jak2/STAT5 signaling system [13]. In addition, 1,25-(OH)_2_D_3_ prolongs GH signaling via the Jak2/STAT5 system in osteoblast-like cells [14].

Methylsulfonylmethane (MSM) is a very simple organic sulfur-containing compound that occurs naturally in a variety of fruits, vegetables, grains, and animals including humans [15]. MSM is a normal oxidative metabolite product of dimethyl sulfoxide (DMSO) that reduces peripheral pain, inflammation and arthritis, and might inhibit the degenerative changes occurring in osteoarthritis [16]. Furthermore, we recently reported that MSM suppresses breast cancer growth by down-regulating STAT3 and STAT5b pathways [17]. This compound can rectify dietary deficiencies and improve cartilage formation [18]. However, the effects of MSM have not yet reported in bone cells and studies of the involvement of MSM in bone differentiation have not been reported. Natural substances have been investigated as candidate materials to be used in bone-related diseases. These natural extracts have been used to develop new drugs through a combination of effective single compounds or in combination with existing commercial drugs, such as estrogen or GH products used to prevent bone loss [19], [20].

In the present study, we investigated whether MSM influences GH signaling via the Jak/STAT pathway in osteoblast-like cells and its underlying molecular mechanism. Furthermore, the effects of MSM on MSC differentiation were investigated. Our results indeed showed that MSM enhanced GH signaling and osteoblast differentiation *via* the Jak2/STAT5b pathway in osteoblast-like cells and MSCs. It is proposed that MSM stimulate longitudinal bone growth and increase osteogenic differentiation via the Jak2/STAT5 pathway.

## Materials and Methods

### Ethics Statement

All procedures for animal experiment were approved by the Konkuk University Institutional Animal Care and Use Committee (Seoul, Korea) on the Use and Care on Animals and performed in accordance with the institutioin guidelines.

### Antibodies and Reagents

Dulbecco's modified eagle's medium (DMEM), modified eagle’s medium (MEM), fetal bovine serum (FBS), and trypsin-EDTA were purchased from Gibco-BRL (Grand Island, NY). IGF-1R [11], phospho-IGF-1R [17], STAT5b [17], Jak2 [13], GHR, tata-binding protein (TBP) antibody [21], and the secondary antibodies (HRP-conjugated goat anti-mouse IgG and HRP-conjugated donkey anti-rabbit IgG) were purchased from Santa Cruz Biotechnology (Santa Cruz, CA). The anti-phosphotyrosine monoclonal antibody 4G10 [11] and phospho-STAT5b [22] were obtained from Upstate Biotechnology (Lake Placid, NY), The anti-actin antibody [11], Methyl sulfonemethane (MSM), Alizarin red S, ascorbic acid phosphate, β-glycerophosphate disodium salt hydrate, L-glutamine, and 3-(4,5-dimethylthiazol-2-yl)-2,5-diphenyl tetrazolium bromide (MTT) were obtained from Sigma Chemical Co. (St. Louis, MO). The SensoLyte *p*NPP Alkaline Phosphatase Assay kit was purchased from ANASREC (San Jose, CA). The luciferase assay substrates and reporter lysis buffer were purchased from Promega Corp. (Madison, WI). The electrophoretic mobility shift assay (EMSA) kit, oligonucleotide probes (STAT5), and nuclear extract kit were purchased from Panomics (Redwood City, CA). FuGene 6 transfection reagent was obtained from Roche (Basel, Switzerland). The RNeasy mini kit was from purchased Qiagen (Hilden, Germany). The reverse transcription-polymerase chain reaction (RT-PCR) Premix kit, IGF-1R, GHR, alkaline phosphatase (ALP), bone sialoprotein (BSP), osteocalcin (OCN), osteonectin (ON), osteopontin (OPN), osterix, Runx2, and 18S primers for RT-PCR were purchased from Bioneer (Daejeon, Korea). The enhanced chemiluminescence (ECL) plus detection kit was purchased from Amersham Pharmacia Biotech. (Piscataway, NJ). The Coomassie Protein Assay kit and Restore Western Blot Stripping Buffer were purchased from Pierce (Rockford, IL).

### Cell Culture and Osteoblast Differentiation

Osteoblast-like UMR-106 cells were obtained from SY Kim [23] and were routinely grown in DMEM medium containing 5% FBS and 4 mM glutamine. MG-63 (No: 21427, KCLB, Korea) cells were cultured in MEM medium containing 10% FBS, 2 mM glutamine, and 100 U/ml penicillin/streptomycin at 37°C in 5% CO_2_. C3H10T1/2 cells (No: 10226, KCLB, Korea), the mesenchymal stem cells established from C3H mouse embryos were purchased from the Korean Cell Line Bank (Seoul, Korea), and maintained in DMEM containing 10% FBS media. Primary bone marrow stromal cells were prepared from 6-week-old BALB/c mice (Orient Bio, Seongnam-Si, Gyenoggi-Do, Korea) long bones (femur and tibia). Bone marrow was flushed out with α-MEM (Gibco, Franklin Lakes, NJ) containing 10% FBS and maintained in α-MEM containing 10% FBS media. On day 6, the cells were seeded, and the medium was supplemented with 10 mM sodium β–glycerophosphate and 50 µg/ml ascorbic acid to initiate osteoblastic differentiation. Medium was replaced every 2–3 days. Cells were cultured in complete medium supplemented with 10 mM sodium β-glycerophosphate and 50 µg/ml ascorbic acid from days 3 to 21 for osteoblast differentiation experiments. Osteoblast differentiation was evaluated after 3, 5, and 7 days for ALP activity, 5 days for ALP mRNA expression, 14 days for osteonectin, and BSP, and 21 days for osteocalcin and osterix.

### MTT Assay

Cell viability was assayed by measuring blue formazan that was metabolized from 3-(4,5-dimethylthiazol-2-yl)-2,5-diphenyl tetrazolium bromide (MTT) by mitochondrial dehydrogenase, which is active only in live cells. One day before drug application, cells were seeded in 96-well flat-bottomed microtiter plates (3,000–5,000 cells/well). MG-63 and UMR-106 cells were incubated for 24 h with various concentrations of MSM. C3H10T1/2 cells and primary bone marrow MSCs were cultured in osteogenic medium (10 mM sodium β-glycerophosphate and 50 µg/ml ascorbic acid) supplemented with various concentrations (0, 5, 10, 15 and 20 mM) of MSM from days 21. Twenty microliters of MTT (5 mg/ml) was added to each well and incubated for 4 h at 37°C. The formazan product was dissolved by adding 200 µl DMSO to each well, and the plates were read at 550 nm. All measurements were performed in triplicate and each experiment was repeated at least three times.

### Western Blot Analysis

MG-63 and UMR-106 cells were treated with the indicated MSM concentrations (0, 5, 10, 15 and 20 mM) for 24 h. Mesenchymal stem cells were cultured in the osteogenic medium with various concentrations (0, 5, 10 and 20 mM) of MSM for 24 h after the initiation of osteoblast differentiation. UMR-106 cells were untreated or pretreated with 50 µM AG490 for 4 h then treated with 20 mM MSM for 24 h. Cells were lysed in whole lysis buffer (50 mM Tris-HCl, pH 7.5, 5 mM EDTA, 150 mM NaCl, and 1% Triton X-100) containing protease and phosphatase inhibitors (1 mM PMSF, 2 µg/ml leupeptin, 4 µg/ml aprotinin, and 1 µg/ml pepstatin), and protein concentrations were determined using the Coomassie Protein Assay (Bradford). An equivalent amount of protein extract from each sample was electrophoresed on 10% SDS-PAGE and transferred to nitrocellulose membranes. Membranes were blocked for 1 h with 5% non-fat milk in T-TBS buffer (20 mM Tris-HCl pH 7.6, 137 mM NaCl, 0.1× Tween20) and incubated overnight at 4°C with primary antibodies (IGF-1R, p-IGF-1R, STAT5b, p-STAT5b, Jak2, GHR and β-actin). The membranes were then washed three times in T-TBS and incubated with the corresponding secondary antibody, anti-mouse or anti-rabbit IgG HRP-conjugate (1∶1000), in T-TBS with 5% non-fat milk for 1 h under agitation at room temperature. After washing three times in T-TBS, the membranes were developed using the ECL PLUS kit.

### Preparation of Whole Cell Extract and Immunoprecipitation

UMR-106 cells were incubated with 50 µM AG490 for 4 h, then left untreated or pretreated with 30 nM GH for 2 h followed by 20 mM MSM for 24 h. Whole cell extracts (WCE) of UMR-106 cells were prepared by lysing the cells in RIPA buffer (50 mM Tris-HCl, pH 7.5, 5 mM EDTA, 150 mM NaCl, and 1% Triton X-100) containing protease and phosphatase (1 mM PMSF, 2 µg/ml leupeptin, 4 µg/ml aprotinin, and 1 µg/ml pepstatin). For immunoprecipitation, 500 µg of WCE was incubated with 4 µl anti-Jak2 antibody for 1 h at 4°C. Protein G agarose beads were added, and the mixture was incubated overnight at 4°C on a rocking platform. The beads were washed four times in 1× IP buffer, resuspended in 2× electrophoresis sample buffer, and boiled for 5 min. The WCE (20 µl per lane) or immunoprecipitated proteins were subjected to SDS-PAGE and electrophoretically transferred onto a nitrocellulose membrane. The Jak2 and STAT5b precipitation and phosphorylation status analysed using Western blot with anti-Jak2, anti-STAT5b, and 4G10 antibodies.

### EMSA

STAT5 DNA binding activity was detected using an EMSA, in which a labeled double-stranded DNA sequence was used as a DNA probe to bind active STAT5b protein in nuclear extracts. Nuclear protein extracts were prepared with a Nuclear Extract kit (Panomics, Freemont, CA). The EMSA was performed by incubating a biotin-labeled transcription factor (TF-STAT5) probe with treated and untreated nuclear extracts. Reactions were resolved on a nondenaturing 6% PAGE gel. Proteins in the gel were transferred to a nylon membrane and detected using streptavidin-HRP and a chemiluminescent substrate.

### Cotransfection and Luciferase Assay

Expression vectors for mouse STAT5b (pMX/STAT5b; kindly provided by Dr. Koichi Ikuta, Kyoto University, Japan) were constructed as described previously [24]. The pGL2P plasmid is an enhancer-less plasmid containing the SV40 promoter (Promega). The 700-bp pGL2P contains the 700-bp distal 5′-flanking region of the IGF-1 gene, identified as a STAT5-binding enhancer, compared with pGL2P (kindly provided by Dr. Honglin Jiang, Virginia Polytechnic Institute and State University, USA). The insert from 700-bp pGL2P was inserted into the pGL2-promoter vector at the Sma1 and Kpn1 sites [24]. The IGF-1R (−2350/+640) LUC reporter plasmid (kindly provided by Dr. Haim Werner, Tel Aviv University, Israel) was subcloned upstream of a promoterless firefly luciferase reporter in the pGL2P vector. The IGF-1R (−2350/+640) LUC reporter plasmid or the 700 bp IGF-1-pGL2 was cotransfected into UMR-106 cells with the STAT5b expression vector, the β-galactosidase expression plasmid (pCMV β-Gal) and either empty pGL2 vector for reporter gene assays. Transfected cells were washed twice with ice-cold PBS, and 150 µl of lysis buffer was added to the wells. Lysates were then used directly to measure luciferase activity. A 100 µl aliquot of cell lysates was mixed with 350 µl of assay buffer containing 25 mM glycylglycine, pH 7.8, 15 mM MgSO4, 4 mM EGTA, 5 mM ATP, and 1 mM DTT. Luciferase activity was determined by measuring luminescence for 10 s on a Lumat LB 9507 luminometer (EG&G Berthold, Oak Ridge, TN) after injecting 100 µl of 1 mM luciferine.

### RT-PCR

UMR-106 cells were untreated or pretreated with 50 µM AG490 for 4 h then treated with 20 mM MSM for 24 h. Bone marrow mesenchymal stem cells were cultured in osteogenic medium and the mRNA expression after the treatment with 20 mM MSM at 5 days for ALP, 14 days for osteonectin, and BSP, and 21 days for osteocalcin and osterix. Total RNA was prepared using the RNeasy Mini kit (Qiagen) and cDNA was synthesized using the AccuPower RT PreMix kit (Bioneer) according to the manufacturer’s instructions. The PCR analysis was performed on aliquots of cDNA to detect IGF-1R (product size: 522 bp), GHR (165 bp), ALP (454 bp), BSP (450 bp), OCN (294 bp), ON (569 bp) and Osterix (160 bp). The PCR primer sequences were as follows: IGF-1R, sense: 5′-ACTATGCCGGTGTCTGTGTG-3′, antisense: 5′-TGCAAGTTCT GGTTGTCGAG-3′, GHR, sense: 5′-TTCTAAACAGCAAAGGATTAA-3′, antisense: 5′-CACTGTGAAATTCGGGTT TA-3′, ALP, sense: 5′-TGGAGCTTCAGAAGCTC AACACCA-3′, antisense: 5′-ATCTCGTTGTCTGAGTACCAGTCC-3′, BSP, sense: 5′-AATGAAAA CGAAGAAAGCGAAG-3′, anti sense: 5′- ATCATAGCCATCGTAGCCTTGT-3′, osteocalcin (OCN), sense: 5′-ATGAGAGCCCTCACACTCCTC-3′, antisense: 5′-GCCGTAGAAGCG CCGATAGGC-3′, osteonectin (ON), sense: 5′-TGGATCTTCTTTCTCCTTT-3′, antisense: 5′-TTCTGCTTCTCAGTCAGA-3′, Osterix, sense: 5′-TAATGGGCTCCTTT CACCTG-3′, anti sense: 5′-CACTGGGCA GACAGT CAGAA-3′. For control purposes, levels of 18S mRNA were measured using the following primer: sense: 5′-CGGCTACCACATCCAAGGAA-3′, antisense: 5′-CCGGCGTCCCCTCTTAATC-3′. The PCR reaction was conducted; 30 cycles at 94°C for 45 seconds, 60°C for 45 seconds, and 72°C for 1 min. After amplification, the PCR products were analyzed on a 1.2% agarose gel and visualized by ethidium bromide staining and ultraviolet irradiation.

### Small Interference RNA (siRNA) Assay

C3H10T1/2 cells were grown to 50% confluence and transfected with ON-TARGETplus SMARTpool *si*RNA targeting STAT5b or ON-TARGETplus Non-targeting *si*RNA (Dharmacon, Chicago) using FuGene 6, according to the manufacturer’s instructions. 48 hours after transfection, cells were cultured with serum free osteogenic medium for 24 h and then cultured in osteogenic medium with 20 mM MSM for 24 h after the initiation of osteoblast differentiation, STAT5b, p-STAT5, IGF-1R, p-IGF-1R and Jak2 expression level was detected using western blotting assay. Osteogenic differentiation marker genes (ALP, BSP, OCN, OPN, Osterix and Runx2) and STAT5b gene expression was analyzed at day 5, 14 and 21 after MSM treatment in C3H10T1/2 cells transfected with STAT5b siRNA or non-target siRNA by real-time PCR.

### Gene Expression by Real-time PCR Analysis

Total RNA was extracted from cultured bone marrow MSCs and C3H10T1/2 cells at different time point (at 5 days for ALP, 14 days for osteonectin, and BSP, and 21 days for osteocalcin and osterix mRNA expression) after the treatment with 20 mM MSM, and the extracted RNA was dissolved in RNase-free distilled water. One microgram of total RNA was first reverse transcribed into cDNA using the AccuPower RT PreMix kit (Bioneer) according to the manufacturer’s instructions. Quantitative PCR was performed in a 20 µl solution, including 1X FastStart DNA Master SYBR (Roche), 25 mM MgCl_2_, diluted gene primers and cDNA. The primer sequences were as follows: ALP, sense: 5′-AATGGGCGTCTCCACAGTAAC-3′, antisense: 5′-CTGAGTGGTGTTGCATCGC-3′, BSP, sense: 5′-CAGAGGAG GCAAGCGT CACT-3′, antisense: 5′-CTGTCTGGGTGCC AACACTG-3′, OCN, sense: 5′-AAGCAGGAGG GCAATAAGGT-3′, antisense: 5′-CAAGC AGGGTTAAGCTCACA-3′, OPN, sense: 5′-TCCAATGAAAGCCATGACCACA-3′, antisense: 5′-TGCTTGTGTACTAGCAGTGACG-3′, osterix, sense: 5′-TAATGGGCTCCTTTC ACCTG-3′, antisense: 5′-CACTGGGCAGAC AGTCAGAA-3′, Runx2, sense: 5′-TCACTACCAGCCACCGAGAC-3′, antisense: 5′-ACGCCATAGTCCCTCCTTTT-3′, STAT5b, sense: 5′-GACTCTGAAATTGGTGGCATCA-3′, antisense: 5′-GATTCCAAAACATTCTTTCCTGAG-3′, GAPDH, sense: 5′-GGGCATCT TGGGCTA CAC-3′, antisense: 5′-GGTCCAGGGTTT CTTACTCC-3′. Assay were performed on a LightCycler (Roche) and analyzed with the accompanying LightCycler software 4.05. The cycling conditions were 40 cycles of two step cycling involving a denaturation step at 95°C for 10 sec and combined annealing/extension step at 60°C for 20 sec. The threshold cycle (Ct) value was calculated from amplification plots. Data were analyzed using the ΔΔCt relative quantification approach. The control calibrator was a pool of reverse transcribed samples with each sample being normalized for internal control GAPDH. Each sample was run in three replicates and was expressed relative to control calibrator at each time point.

### ALP Activity

ALP assay was performed on days 3, 5 and 7 differentiation by biochemical colorimetric assays in ALP kits as directed by the manufacturer. Cells were plated in triplicate on 96-well plates, incubated with MSM at varying concentrations (0, 5, 10 and 20 mM) including osteogenic medium, and add with 100 µl of ALP substrate solution (SensoLyte *p*NPP Alkaline Phosphatase Assay kit) for 40–60 min at room temperature. The reaction was stopped with stop buffer, and the absorbance at 405 nm was measured as the ALP activity using a microplate reader. ALP activity is expressed as p-nitrophenol production per total protein. Protein concentration was determined using the Coomassie Protein assay reagent (Bradford assay), with bovine serum albumin as the standard.

### Mineralization Assay

Alizarin Red staining and von Kossa staining of the MSC cultures was carried out at day 21 after the MSM treatment to evaluate the effect of MSM on the matrix mineralization of MSCs. The cells were washed with PBS, fixed with 4% paraformaldehyde in PBS for 15 min, and rinsed with distilled water three times. For alizarin res S staining, 40 mM alizarin red S (pH 4.2) stain was added to the plates and incubated for 10 min at room temperature, rinsed three times with distilled water, and finally washed with PBS to reduce non-specific staining. For von Kossa staining, the fixed cells were stained with freshly prepared 5% silver nitrate under UV light for 1 h to detect phosphate deposits. Background color was removed by 5% sodium thiosulfate, and the cells were rinsed three times with distilled water. They were observed using phase contrast microscopy (Olympus DP71); images were captured using DPC controller software. Photographs were taken using a Nikon digital camera.

### Statistical Analysis

All data values were expressed as mean ± SEM. Statistical analysis was done with student’s t-test or ANOVA test of the SAS program. These were compared by one-way analysis of variance (ANOVA) followed by Duncan’s multiple range test. A value of P<0.05 was considered a significant difference.

## Results

### MSM Cytotoxicity of Osteoblast-like Cells

**Figure 1:**
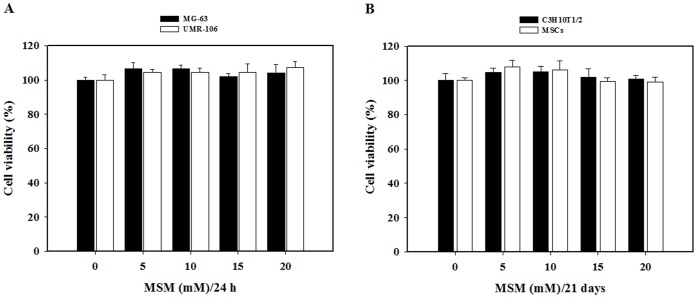
Effects of methylsulfonylmethane (MSM) on viability in osteoblast-like cells and MSCs. MG-63 and UMR-106 cells exposed to control condition without MSM or growth facilitated condition with increasing concentration of MSM for 24 h. C3H10T1/2 cells and mesenchymal stem cells grown in the osteogenic media (10 mM sodium β-glycerophosphate and 50 µg/ml ascorbic acid) and exposed to control condition or growth facilitated condition for 21 days. After culture, cell viability was evaluated using MTT assay. Data shown are representative of three independent experiments.

The MG-63 and UMR-106 osteoblast-like cells were treated with a various concentrations of MSM (0, 5, 10, 15, or 20 mM) for 24 h to investigate MSM cytotoxicity of osteoblast-like cells. We first examined whether MSM was cytotoxic to osteoblast-like cells. No notable cytotoxicity was observed when the cells were exposed to up to 20 mM for 24 h (Fig. 1A). Also, C3H10T1/2 cells and primary bone marrow MSCs were cultured with various concentrations of MSM (0, 5, 10, 15, or 20 mM) for 21 days. As shown in osteoblast-like cells, C3H10T1/2 cells and MSCs had no cytotoxic effects (Fig. 1B). Thus, we used MSM at a concentration of 0–20 mM for subsequent experiments.

### MSM Increases GH Signaling-related Protein Expression in Osteoblast-like Cells and MSCs

**Figure 2:**
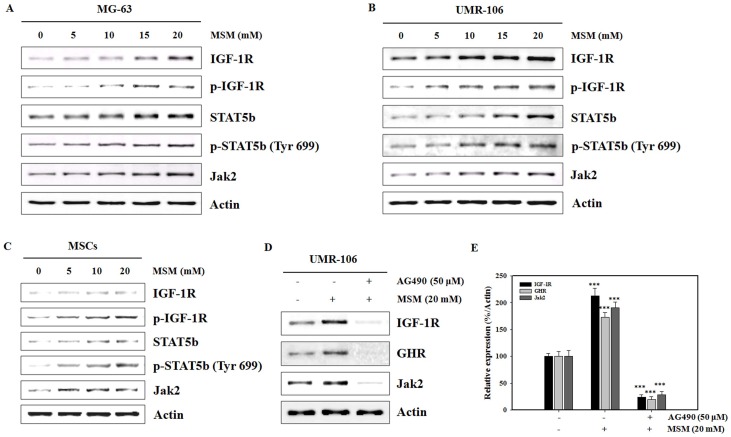
Effects of methylsulfonylmethane (MSM) on the expression of growth hormone (GH) signaling-related proteins in osteoblast-like cells and MSCs. MG-63 (A) and UMR-106 (B) cells were treated with the indicated MSM concentrations for 24 h. (C) Mesenchymal stem cells were cultured in osteogenic medium with various concentrations of MSM for 21 days. (D) UMR-106 cells were left untreated or pretreated with 50 µM AG490 for 4 h then treated with MSM for 24 h. Protein extracts (20 µg) were separated by 10% SDS-PAGE, and Western blots were performed. β-actin was used as a protein loading control. (E) The relative levels of IGF-1R, GHR and Jak2 protein were determined using densitometric analysis and normalized to the amount of β-actin. This picture is representative of three independent experiments. Asterisks indicate a statistically significant increase by ANOVA (***p<0.001).

The expression levels of various proteins involved in GH signaling were assessed by Western blotting. GH signals by binding to the GHR to activate tyrosine kinase, Jak2, and downstream pathways including STAT5, thereby regulating gene expression such as IGF-1. GH exerts effects both directly and via IGF-1, which signals by activating the IGF-1R. We hypothesized that MSM increases the expression of IGF-1R, phospho-IGF-1R, STAT5b, Jak2, and phosphorylation of STAT5b in osteobast-like cells (MG-63 and UMR-106) and primary bone marrow MSCs. As shown in Fig. 2A, B, and C, MSM treatment dose-dependently increased expression of IGF-1R, phospho-IGF-1R, STAT5b, Jak2, and phosphorylation of STAT5b in the three cell lines. These finding suggest that MSM involves the Jak2/STAT5b signaling pathway in MSCs. We next checked inhibition of Jak2 by AG490 which lead to a blockade of MSM-induced IGF-1R and GHR protein expression. MSM-induced IGF-1R and GHR protein expression was inhibited by AG490 (Fig. 2D). The relative expression density of protein with respect to actin gave a clear view on the effect of MSM on UMR-106 cells at AG490 (Fig. 2E). These results suggest that MSM-induced signaling is similar to GH signaling via the Jak2/STAT5 pathway.

### Inhibition of MSM-induced IGF-1R and GHR Expression by the Jak2 Inhibitor AG490 in UMR-106 Cells

**Figure 3:**
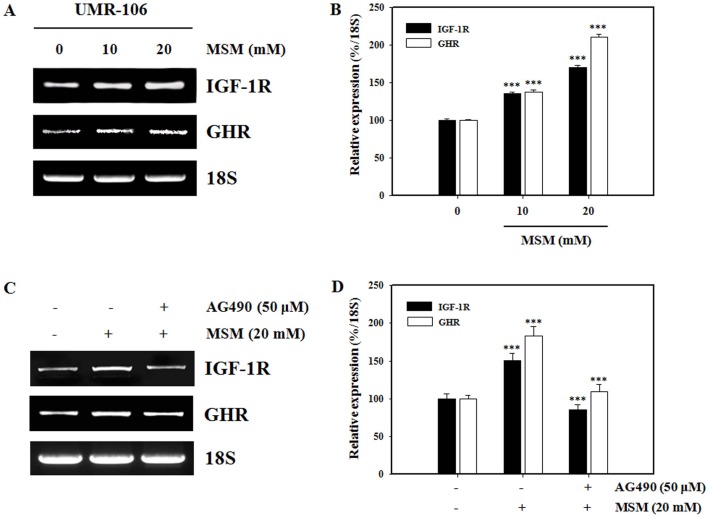
Methylsulfonylmethane (MSM) activates the expression of growth hormone (GH) signaling-related mRNA in UMR 106 cells. Total RNA was isolated from the UMR-106 cells using an RNeasy kit. The cDNA was amplified using specific primers for insulin like growth factor-1 receptor (IGF-1R), the growth hormone receptor (GHR) or 18S. 18S was used as a control. (A) UMR-106 cells were treated with the indicated concentrations of MSM for 24 h. (B) The relative levels of IGF-1R and GHR mRNA were determined using densitometric analysis and normalized to the amount of 18S. (C) UMR-106 cells were left untreated or pretreated with 50 µM AG490 for 4 h then treated with MSM for 24 h. (D) The relative levels of IGF-1R and GHR mRNA were determined using densitometric analysis and normalized to the amount of 18S. Data shown are representative of three independent experiments. Asterisks indicate a statistically significant increase by ANOVA (***p<0.001).

We examined the effect of Jak2 inhibition on MSM-induced IGF-1R and GHR expression. As shown in Fig. 3A, MSM upregulated IGF-1R and GHR mRNA expression in a dose dependent manner. The relative expression density of mRNA with respect to 18S gave a clear view on the effect of MSM on UMR-106 cells at different concentration levels (Fig. 3B) and AG490 (Fig. 3D). We next checked inhibition of Jak2 by AG490 which lead to a blockade of MSM-induced IGF-1R and GHR mRNA expression (Fig. 3C). These results suggest that MSM-induced IGF-1R and GHR expression increases through Jak2/STAT5b.

### MSM Induces STAT5 Binding to the IGF-1R Promoter Site

**Figure 4:**
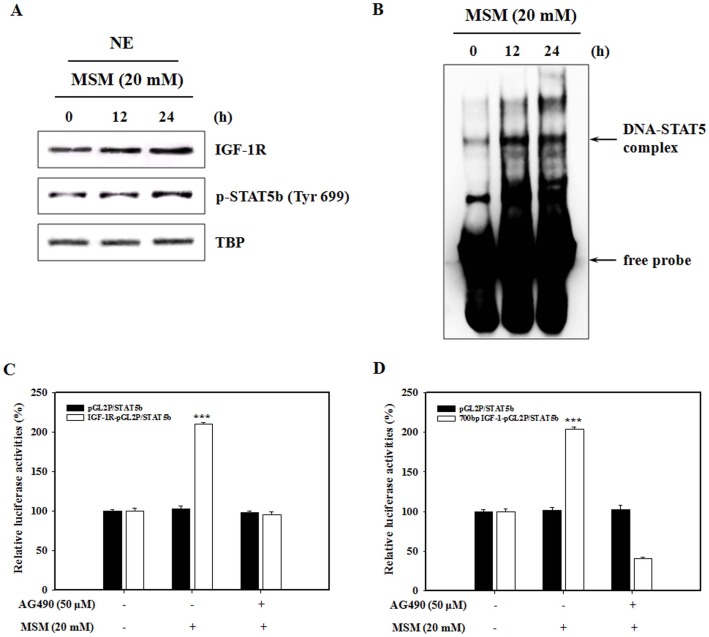
Methylsulfonylmethane (MSM)-induced binding activity of STAT5b to the insulin-like growth factor-1 receptor (IGF-1R) GAS site. (A) UMR-106 cells were cultured in serum-free MEM for 24 h and incubated with MSM (20 mM) for 12 or 24 h. Nuclear extracts (NE) were separated and blotted onto a nitrocellulose membrane, which showed an increase in the level of IGF-1R and STAT5b. Tata binding protein (TBP) was used as a nuclear protein loading control. (B) STAT5b DNA binding was detected by electrophoretic mobility shift assay. Nuclear extracts were prepared and incubated with STAT5 probe (from a part of Panomics EMSA kits). The resulting complexes were electrophoresed on a 6% non-denaturing gel. (C) The IGF-1R (−2350/+640) LUC reporter plasmid or (D) the 700 bp IGF-1-pGL2 were cotransfected into UMR 106 cells with the STAT5b expression vector, the β-galactosidase expression plasmid (pCMV β-Gal), and either empty pGL2 vector, and then incubated with 50 µM AG490 for 4 h, then treated with 20 mM MSM for 24 h. Promoter activities were expressed as luciferase normalized to β-galactosidase values. Data shown are representative of three independent experiments. Asterisks indicate a statistically significant increase by ANOVA (***p<0.001).

Translocation of initiated STATs to the nucleus forms dimers and binds to specific response elements in the promoters of target genes, and transcriptionally activates these genes. The STAT nuclear translocation and DNA binding activity are probably not influenced by STAT serine phosphorylation, but they are influenced by STAT tyrosine phosphorylation [25]. GH was able to induce phosphorylation and DNA binding of STAT5 in UMR-106 cells [11]. Western blotting and the EMSA were carried out to examine the effects of MSM on p-STAT5b nuclear protein level and STAT5 DNA-binding activity in UMR 106 cells. A significantly increased p-STAT5b level was detected in nuclear extracts from cells treated with MSM (Fig. 4A). Furthermore, MSM increased binding to the IGF-1R promoter sites. STAT5 DNA-binding activity was evident after 12 h of MSM treatment and was maintained for 24 h of stimulation (Fig. 4B). These results suggest that MSM mainly induces specific phosphorylation and DNA-binding activity of STAT5 in the absence of GH. Hence, MSM increased the phosphorylation of STAT5b to p-STAT5b, and binding to the promoter site of IGF-1R. Osteoblasts express IGF-1R and can thus respond to IGF-1. IGF-1 stimulates osteoblast proliferation, collagen stability, and bone mineralization [26], [27]. Thus, we evaluated the transcriptional effects of MSM on IGF-1R/STAT5b and IGF-1/STAT5b. The GH-related reporter construct IGF-1R (−2350/+640) the LUC reporter plasmid, or the 700 bp IGF-1-pGL2 was cotransfected into UMR 106 cells with the STAT5b expression vector. No increase in luciferase activity was detected when the empty pGL2 vector was transfected with the STAT5b expression vector (Fig. 4C and D). In contrast, relative luciferase activity increased after 24 h of MSM treatment and the difference was significant for STAT5b/IGF-1R and STAT5b/IGF-1 (***P<0.001) (Fig. 4C and D). The involvement of STAT5b in MSM-induced transcriptional activation of the IGF-1R or IGF-1 was confirmed using the Jak2 inhibitor AG490, which is well known to inhibit the Jak2/STAT5 pathway [28]. These observations suggest that MSM increases IGF-1R and IGF-1 expression via STAT5b activation.

### MSM Enhances GH Signaling via Jak2/STAT5b Activation in UMR 106 Cells

**Figure 5:**
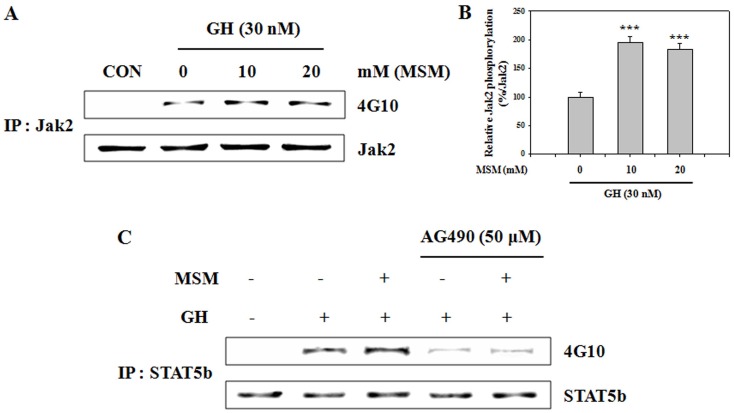
Methylsulfonylmethane (MSM) enhances growth hormone (GH) signaling via Jak2/STAT5b activation in osteoblast-like cells. (A) Jak2 was immunoprecipitated with anti-Jak2 antibody from whole cell extracts of UMR 106 cells left untreated or pretreated with 30 nM GH for 2 h followed by MSM for 24 h. Jak2 precipitation and the phosphorylation status of Jak2 were analyzed by Western blotting with anti-Jak2 and anti-phosphotyrosine (4G10) antibodies. (B) The relative levels of Jak2 phosphorylation were determined using densitometric analysis and normalized to the amount of Jak2. (C) UMR-106 cells were incubated with 50 µM AG490 for 4 h, then left untreated or pretreated with 30 nM GH for 2 h followed by MSM for 24 h. STAT5b precipitation and the phosphorylation status of the precipitated STAT5b were analyzed by Western blot with anti-STAT5b and 4G10 antibodies. This picture is representative of three independent experiments. Data shown are representative of three independent experiments. Asterisks indicate a statistically significant increase by t-test (***p<0.001).

According to a recent study, the osteoblast-like osteosarcoma cell line UMR 106 expresses a GH-responsive Jak2/STAT5 signaling system [29]. We investigated whether MSM influences GH signaling via the Jak2/STAT5 pathway in UMR 106 cells. Whole cell extracts were immunoprecipitated with anti-Jak2 antibody. Immunoprecipitates were analyzed by Western blot using anti-4G10 antibody. A dose-dependent increase in Jak2 phosphorylation was detected in GH-pretreated and MSM-treated UMR 106 cells, compared with cells not pretreated with GH. Interestingly, Jak2 phosphorylation increased further in GH-pretreated and MSM-treated cells as compared with GH-treated cells (Fig. 5A). These results demonstrate that MSM enhanced GH-induced Jak2 activation. We next analyzed the effect of MSM on GH-induced STAT5b activation. UMR-106 cells were incubated with 50 µM AG490 for 4 h, then left untreated or pretreated with 30 nM GH for 2 h followed by MSM treatment for 24 h. The phosphorylation status of the precipitated STAT5b was analyzed by Western blot with anti-phospho-STAT5b (Y699) antibodies. STAT5b phosphorylation was further increased in GH-pretreated with MSM-treated cells compared with that in GH-pretreated cells (Fig. 5C). The inhibition of Jak2 by AG490 lead to a blockade of MSM treatment on GH-induced STAT5b phosphorylation. These findings suggest that MSM-enhanced GH signaling requires the Jak2/STAT5 activation in UMR 106 cells.

### MSM-enhanced GH Signaling Requires STAT5b Activation in C3H10T1/2 Cells

**Figure 6:**
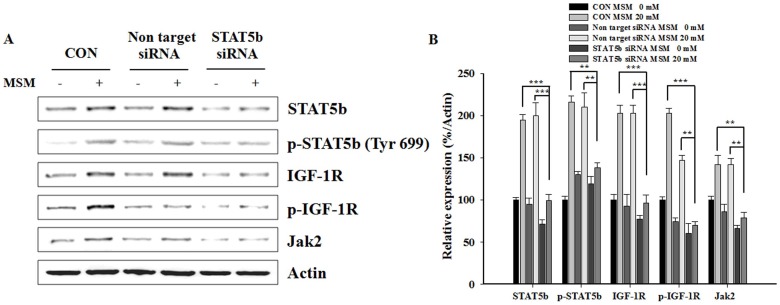
Methylsulfonylmethane (MSM)-enhanced GH signaling requires STAT5b activation in C3H10T1/2 cells. (A) C3H10T1/2 cells were grown to 50% confluence and transfected with ON-TARGETplus SMARTpool siRNA targeting STAT5b or ON-TARGETplus Non-targeting siRNA using FuGene 6, according to the manufacturer’s instructions. 48 hours after transfection, cells were cultured with serum free osteogenic medium for 24 h and then cultured in osteogenic medium with 20 mM MSM for 24 h after the initiate osteoblast differentiation. Protein extracts (20 µg) were separated by 10% SDS-PAGE, and Western blots were performed. β-actin was used as a protein loading control. (B) The relative levels of protein were determined using densitometric analysis and normalized to the amount of β-actin. Data shown are representative of three independent experiments. Asterisks indicate a statistically significant increase by t-test (**p<0.01, ***p<0.001).

To explore whether STAT5b is involved in effects of MSM on GH signaling, we used siRNA strategy to examine the effect of STAT5b-related GH signaling on MSM-mediated osteogenesis. C3H10T1/2 cells were transfected with specific STAT5b siRNA and then exposed to MSM treatment. STAT5b knockdown decreased the basal level of STAT5b protein expression. Knockdown of STAT5b also inhibited MSM-induced phospho-STAT5b, IGF-1R, phospho-IGF1-R, and Jak2 expression level in C3H10T1/2 cells (Fig. 6A). The relative expression density of protein with respect to actin gave a clear view on the effect of STAT5b-related GH signaling on MSM-mediated osteogenesis. (Fig. 6B). These results demonstrated that STAT5b played an essential role in GH signaling activation in C3H10T1/2 cells.

### Involvement of STAT5b in MSM-induced Osteogenic Marker Genes of MSCs

**Figure 7:**
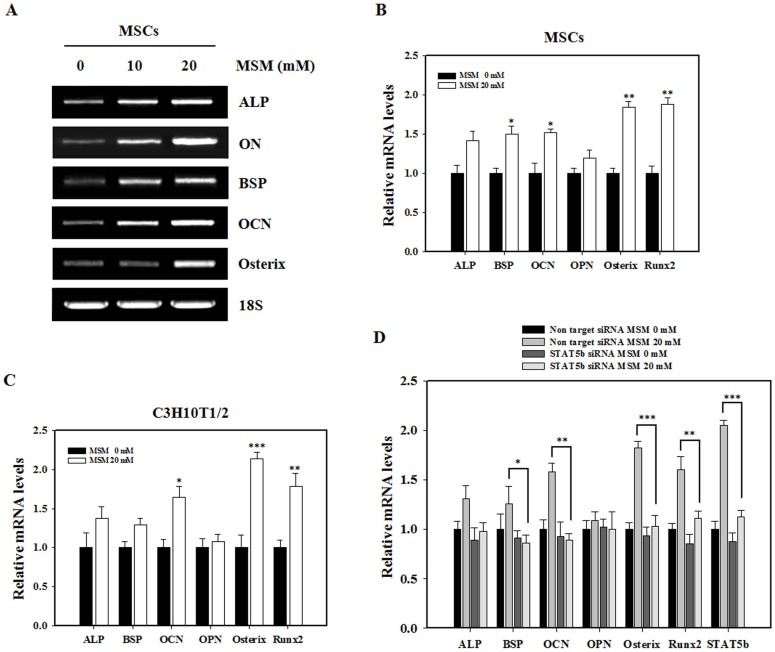
Involvement of STAT5b in MSM-induced osteogenic marker genes in MSCs. (A) Bone marrow mesenchymal stem cells were cultured in the osteogenic medium at 5 days for ALP, 14 days for osteonectin (ON) and bone sialoprotein (BSP), and 21 days for osteocalcin (OCN) and osterix mRNA expression after the treatment with various concentrations (0, 10 and 20 mM) of MSM. RT-PCR was performed using the cDNA and primers for ALP, ON, BSP, OCN, osterix and 18S. Total RNA was isolated from the MSCs using an RNeasy kit. 18S was used as a control. (B) Bone marrow Mesenchymal stem cells and (C) C3H10T1/2 cells were cultured in osteogenic medium at 5 days for ALP and Runx2, 14 days for OPN and BSP, and 21 days for OCN and osterix mRNA expression after the treatment with 20 mM MSM. After culture, real-time PCR was performed. (D) Osteogenic differentiation marker genes (ALP, BSP, OCN, OPN, Osterix and Runx2) and STAT5b gene expression was analyzed at day 5, 14 and 21 after MSM treatment in C3H10T1/2 cells transfected with STAT5b siRNA or non-target siRNA. The effect of STAT5b knockdown on osteogenic marker genes was analyzed by real-time PCR. GAPDH was used as the internal control. The relative levels of mRNA were determined using densitometric analysis and normalized to the amount of GAPDH. Data shown are representative of three independent experiments. Asterisks indicate a statistically significant increase by t-test (*p<0.05, **p<0.01, ***p<0.001).

We analyzed the effect of MSM on the expression of five classical osteoblastic marker genes, namely ALP, ON, BSP, OCN, and Osterix by RT-PCR. The mRNA level of osteogenic-specific markers was dose-dependently increased by MSM in primary bone marrow MSCs (Fig. 7A). Not only early-stage osteogenic differentiation marker (ALP), but also middle- and late-stage osteogenic differentiation markers (ON, BSP, OCN, and Osterix), were positively affected by MSM. In addition, we have analyzed the effect of MSM on the expression of osteoblastic marker genes in primary bone marrow MSCs and C3H10T1/2 cells using real-time PCR. As shown in Fig. 7B and C, MSM significantly increased OCN, Osterix, and Runx2 gene expression in primary bone marrow MSCs and C3H10T1/2 cells. To explore the role of STAT5b in MSM-induced osteogenic marker genes activation, we used STAT5b siRNA and non-target siRNA. C3H10T1/2 cells were transfected with specific STAT5b siRNA and then exposed to MSM treatment. Consistent with the diminished GH-related proteins expression (Fig. 6A), STAT5b knockdown significantly reduced MSM-induced up-regulation of osteogenic marker genes (OCN, Osterix, and Runx2) and STAT5b gene (Fig. 7D). All together, these results demonstrated STAT5b activation and subsequent osteogenic differentiation in response to MSM treatment in C3H10T1/2 cells.

### MSM Promotes Osteogenic Differentiation of Mesenchymal Stem Cells

**Figure 8:**
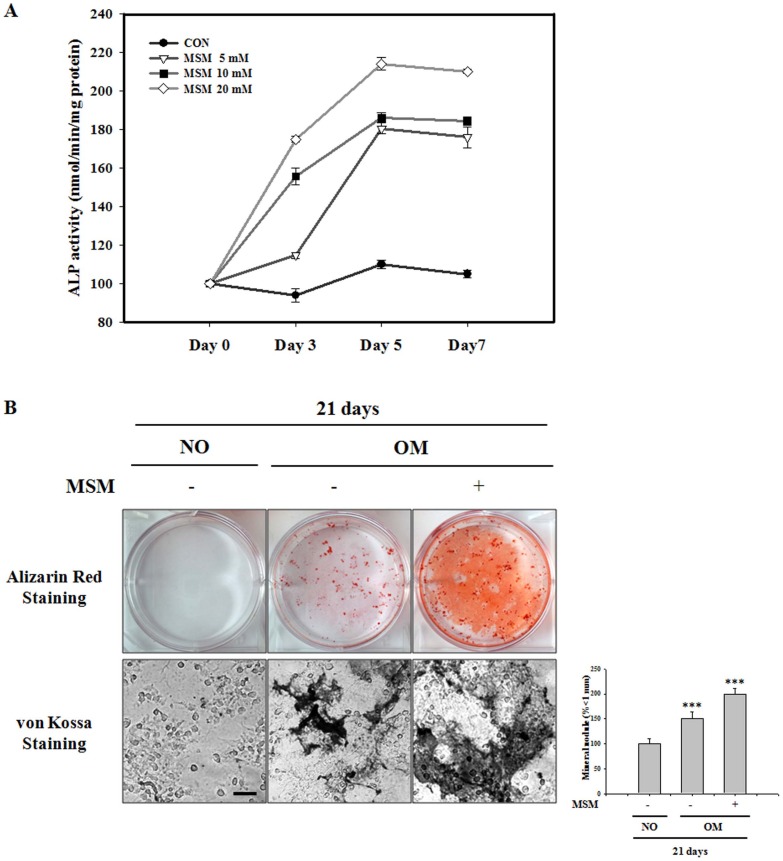
Effects of methylsulfonylmethane (MSM) on osteoblast differentiation in primary murine bone marrow mesenchymal stem cells (MSCs). (A) Comparison of alkaline phosphatase (ALP) activity of various MSM treatments with varying culture durations. Cells were treated with various concentrations (0, 5, 10 and 20 mM) of MSM for 3, 5, and 7 days. ALP activity was measured by the release of p-nitrophenol from p-nitrophenyl phosphate and was normalized to the protein level. Osteoblastic mineralization was determined by Alizarin Red S staining (B) and von Kossa staining (C) at day 21. Cells were cultured in non osteogenic medium (NO) or osteogenic medium (OM) with various concentrations (0 and 20 mM) of MSM for 21 days (scale bar: 50 µm). Mineral nodules (more than 1 mm in diameter) were counted and expressed in histogram. Data shown are representative of three independent experiments. Asterisks indicate a statistically significant increase by ANOVA (***p<0.001).

We studied the effects of MSM on differentiation in bone marrow MSCs. Cells were cultured with various concentrations of MSM for 3, 5, and 7 days. ALP activity, an early-stage osteogenic differentiation marker, increased significantly at 5 days of culture, with dose-dependency for MSM. The control group had significantly less activity compared to that in all treated experiments (Fig. 8A). These mesenchymal cells differentiated into osteoblasts in the presence of osteoblast differentiation medium, and the matrix started to mineralize. To determine osteoblastic mineralization, bone marrow MSCs cells were cultured with 20 mM MSM in osteogenic medium for 21 days. We used the Alizarin Red S staining to visualize the precipitated calcium incorporated into the matrix. As Alizarin Red S stains intra-cellular calcium as well as calcium binding proteins and proteoglycans, it is useful to evaluate the early phases of differentiation [30]. Extracelular calcium deposition by mature osteoblasts was confirmed by von Kossa staining, which detected phosphate of calcium phosphate of secreted matrix. Cells undergoing osteoblast differentiation and mineralization provided positive staining, enabling comparison in the third week of differentiation. As shown in Fig. 8B, MSM dramatically increased the mineralized area visualized by Alizarin Red S staining for calcium. Similar results were obtained after von Kossa staining. Cells of untreated plates in non-osteogenic medium (NO) were negative for Alizarin red S and von Kossa staining even 21 days of culture. The other hand, cells of untreated plates in osteogenic medium (OM) were positive for Alizarin red S and von Kossa staining even 21 days of culture. These results suggest that MSM may induce osteogenic differentiation throughout the process from the early to the late phase.

## Discussion

MSM is a popular dietary supplement used for a variety of conditions including pain, inflammation, allergies, arthritis, parasitic infections, and maintenance of normal keratin levels in hair, skin, and nails [31]. In our recent study, we found that MSM suppresses tumor growth via inhibition of the STAT3 and STAT5b pathways, while being non-toxic to normal cell line MCF-10A [17]. Same way in another study conducted by Caron et al., in 2010 showed MSM induces loss of metastatic properties and reemergence of normal phenotype in murine melanoma cell line [32]. The potential toxicity of MSM has been extensively tested. These studies concluded that MSM is essentially non-toxic to humans [32]. The cell proliferation assay using MTT method demonstrated that MSM at 5, 10, 15, and 20 mM exerted a dose dependent effect on the proliferation of osteoblast-like cells and MSCs. As shown in Fig. 1, there was no significant difference on the OD values in osteoblast-like cells and MSCs. Despite its popularity and non-toxicity, no published bone metabolism data are available on MSM in osteoblasts.

We investigated the influence of MSM on GH signaling via the Jak/STAT pathway in osteoblast-like cells. GH-induced Jak2 activation resulted in the engagement of several intracellular signaling pathways, including STATs −1, −3, and 5 [33], [34]. Activation of STAT5b is prominent in GH signaling and is critical for regulating several GH-responsive IGF-1 genes [35], [36]. We found that MSM enhanced GH-induced Jak2 and STAT5b activation in UMR-106 osteoblast-like cells. MSM increased the expression of GH signaling-related proteins including IGF-1R, p-IGF-1R, STAT5b, p-STAT5b, and Jak2 in osteoblast-like cells (MG-63 and UMR-106) and primary bone marrow MSCs (Fig. 2). The UMR 106 is a rat clonal cell line with well-characterized osteoblast-like phenotypic properties such as endogenous GHR expression [13]. Osteoblasts express GHR [37]–[39], and GH induces a GHR-Jak2-IGF-1R complex, suggesting a novel function for IGF-1R [40]. We have also shown that MSM increased the expression of IGF-1R and GHR mRNA in osteoblast-like cells. The expression of MSM-induced IGF-1R and GHR was inhibited by AG490, a Jak2 kinase inhibitor (Fig. 3). Our data suggest that MSM-induced signaling is similar to GH signaling via the GHR-Jak2.

In a recent study by Gerland et al. [29] GH activated Jak2 and STAT5, but not STAT5 transcriptional activity in UMR-106 cells. They showed that GH could induce a transcriptional response in STAT5 or GH receptor-transfected UMR-106 cells [14]. In our study, we detected MSM-induced STAT5 DNA binding activity in the absence of GH. Interestingly, MSM increased STAT5 binding to the IGF-1R promoter site (Fig. 4B). GH-dependent IGF-1 gene expression is believed to be mediated largely by STAT5 activation via enhancer elements that bind STAT5 [35], [41]. STAT5b is a direct mediator of IGF-1 gene expression produced by hypoxia [24]. We confirmed that MSM increased IGF-1 and IGF-1R promoter activities. The involvement of STAT5b in the MSM-induced transcriptional activation of the IGF-1R or IGF-1 was confirmed using the Jak2 inhibitor AG490. These data showed that MSM increases IGF-1R and IGF-1 expression via STAT5b activation as in GH signaling (Fig. 4C and D).

GH promotes longitudinal growth and regulates multiple cellular functions in humans and animals [11]. In UMR 106 cells, sustained Jak2 tyrosine phosphorylation was observed after GH treatment. Therefore, we investigated whether MSM influenced GH signaling via the Jak2/STAT5b pathway in UMR-106 cells by immunoprecipitation and Western blot. We found that MSM-enhanced GH signaling requires the Jak2/STAT5 activation in UMR 106 cells (Fig. 5). From these results we propose that MSM and GH, separately or in combination, activated GH signaling via the Jak2/STAT5b pathway in UMR-106 cells. Some earlier *in vitro* cell studies showed that co-administration as compared with separate administration of 1,25-(OH)_2_D_3_ and GH was more effective for increasing the expression of osteoblastic marker genes [14], [42], [43]. In agreement with these notions, this study showed that STAT5b is critical for the effect of MSM on C3H10T1/2. STAT5b activity in C3H10T1/2 is diminished with siRNA targeting strategy and inhibited MSM-induced phospho-STAT5b, IGF-1R, phospho-IGF-1R, and Jak2 expression level (Fig. 6). These results indicate that the STAT5b activation plays an essential role in MSM-induced GH signaling of C3H10T1/2 cells.

Based on these findings, we hypothesized that MSM can regulate bone growth and bone metabolism in MSCs. We found that MSM significantly promoted osteoblast differentiation of MSCs. This is a first report that has evaluated the osteogenic potential of MSM. Bone formation is a spatial cascade process, which initiates from the differentiation of bone marrow MSCs into osteo-progenitor cells and then preosteoblasts and osteoblasts, followed by matrix maturation and matrix mineralization [5]. We were able to evaluate osteogenic potential using various parameters such as osteoblast differentiation-associated gene expression levels, the ALP assay, and histochemical staining for minerals.

Among osteogenic markers, MSM increased ALP, ON, BSP, OCN, Osterix, and Runx2 gene expression, although the effects of OPN expression were not detected (Fig. 7A∼C). During the differentiation towards mature osteoblasts, a number of extracellular matrix genes are expressed by MSCs, such as BSP and ON, which is regarded as the middle stage gene marker of osteogenesis [44]. ON is a bone-specific protein that binds selectively to collagen and hydroxyapatite and helps in active mineralization [45]. At the late stage of bone formation, OCN is up-regulated, which represents the maturation of osteoblasts and matrix mineralization processing [44]. Osterix is the downstream mediator for Runx2 actions and the key transcription factor required for differentiation of preosteoblasts to mature osteoblasts [46]. Runx2 regulates its target gene such as OPN, BSP and OCN by binding and transactivating the promoter region. OPN is a direct downstream target of Runx2. Despite Runx2 expression increased by MSM, OPN was not affected by MSM. Runx2 regulatory role in regulating target genes is not always positive, negative regulation by Runx2 has also been reported [47]. These results evoke that MSM effects throughout the osteoblast differentiation. In addition, suppression of STAT5b signaling by siRNA abrogated the enhancement of MSM-induced osteogenic marker genes in C3H10T1/2 cells (Fig. 7D). The data therefore suggest that STAT5b involves in MSM-mediated osteoblastic differentiation of MSCs.

ALP is an early marker and one of the most frequently used markers to demonstrate osteoblast differentiation [48], [49]. ALP in osteoblasts removes pyrophosphate ions, which are potent mineralization inhibitors, to induce mineralization [50]. ALP activity increased significantly at 5 days of MSCs cell culture, with a dose-dependent effect of MSM (Fig. 8A). Interestingly, levels of ALP mRNA expression on MSM provided similar results. The final phase of osteoblast differentiation is mineralization, in which a mineral matrix containing mainly calcium phosphate in the form of hydroxyapatite, is secreted and deposited by mature osteoblasts [51]. In our study, matrix deposition was initiated from day 21 of osteoblast differentiation in cells treated with MSM. We used Alizarin Red S and von Kossa to analyze mineral matrix deposition. Alizarin Red S and von Kossa staining confirmed the marked enhancement of mineralization by cells undergoing osteoblast differentiation on MSM (Fig. 8B). Our osteoblast differentiation data on MSM demonstrated better mineralization, which is essential for bone healing. The up-regulated expression of osteogenic marker genes as well as increased ALP activity and matrix mineralization suggested that MSM has the positive effect on osteogenic differentiation of MSCs.

In conclusion, we suggest that MSM treatment activated GH signaling *via* the Jak2/STAT5b pathway in osteoblast-like cells and MSCs. Also, MSM can promote the osteogenic differentiation of MSCs through the activation of STAT5b in MSCs. In this present study, MSM mimicked the role of GH and intensified the growth of bone and differentiation of osteoblasts. Although further studies are required to clarify the *in vivo* actions and mechanisms, MSM may become a drug candidate for treating bone-depleting diseases, as it is expected to not only enhance bone formation but also suppress bone resorption. Future clinical studies will determine whether some patients with decreased bone mass due to other reasons will benefit from treatment with MSM alone or in combination with GH.
